# Another Call for RCTs of Interventions to Reduce Particulate Matter 2.5 Associated Cardiovascular Health Effects*

**DOI:** 10.1016/j.jacadv.2023.100317

**Published:** 2023-05-26

**Authors:** Wayne E. Cascio, Cavin Ward-Caviness

**Affiliations:** Center for Public Health and Environmental Assessment, Office of Research and Development, U.S. Environmental Protection Agency, Durham, North Carolina, USA

**Keywords:** air pollution, cardiovascular disease prevention, environmental cardiology

In this issue of *JACC: Advances*, Motairek et al^1^ report that chronic (long-term) exposure to inhaled ambient airborne particulate matter <2.5 μm in diameter (PM_2.5_) increases the risk of major adverse clinical events (myocardial infarction, stroke, or all-cause mortality) and life years lost in a cohort of U.S. veterans after elective percutaneous coronary interventions. This study contributes to the extensive literature on cardiovascular health risks of long-term PM_2.5_ exposure and adds new evidence that expands the list of clinical subgroups at risk of poor cardiovascular outcomes associated with long-term exposure to PM_2.5_, as well as highlights the complexity of data acquisition and management, analytical and statistical methods attendant to contemporary environmental epidemiological research, and the challenge of providing evidence-based clinical guidance for health-care professionals. However, with scientific evidence indicating a clear causal relationship between PM_2.5_ exposure and cardiovascular outcomes,^2^ this study and others also showcase disparities between scientific evidence and clinical practice. In short, why are so few at-risk patients being counseled by their health-care professionals about the risks of short- and long-term PM_2.5_ exposure?

The research findings of Motairek et al^1^ are built on established literature documenting the cardiovascular health risk of long-term PM_2.5_ exposure among individuals with pre-existing heart and vascular disease, while ostensibly highlighting an opportunity for risk modification through reduction of exposure to PM_2.5_. Prior research showed that long-term PM_2.5_ exposure increases the risk of cardiovascular mortality, myocardial infarction and stroke in patients with a history of myocardial infarction and stroke.^3^ The health risks go beyond this; however, with other cardiac and renal conditions also observed to have outcomes worsened by PM_2.5_ exposure. In patients with heart failure, Ward-Caviness et al^4,5^ showed that long-term PM_2.5_ exposure was positively associated with mortality and rehospitalization. An increased risk for rehospitalization has also been shown for short-term (daily) PM_2.5_ exposures for individuals with heart failure^6^ and end-stage renal disease.^7^

The U.S. Environmental Protection Agency (EPA) concluded that a causal relationship exists between both short- and long-term PM_2.5_ exposure and cardiovascular effects in their Integrated Science Assessment^2^ of the collective body of scientific evidence (ie, experimental and epidemiologic). Yet, in spite of the strong epidemiologic evidence supporting an association between short-term and long-term exposure to ambient PM_2.5_ and adverse cardiovascular outcomes,^8^ relatively few health-care professionals report talking to their patients with cardiovascular disease about the risk air pollution poses to their health. Mirabelli et al^9^ reported that only 23% of family medicine physicians and 18.7% of internists spoke to their patients with cardiovascular disease about these risks. In another survey, only 3% of patients said that they had discussed approaches to reduce exposure to air pollution with their health-care provider.^10^

The epidemiological, clinical, and toxicological evidence for cardiovascular effects provides some of the strongest scientific justification for the US EPA’s National Ambient Air Quality Standard for Particulate Matter (PM NAAQS), as required under the Clean Air Act, to protect public health including cardiovascular health. However, the PM NAAQS and the US EPA’s Air Quality Index, which is the tool used to convey local air quality information to the public and recommend actions to reduce exposures, do not translate easily into clinical guidance for actions that individuals can take to protect their cardiovascular health. Even the *Million Hearts 2027* initiative, co-led by the Centers for Disease Control (CDC) and the Center for Medicare & Medicaid Services recognizes PM_2.5_, along with increasing physical activity and smoking cessation, as important modifiable factors for “Building Healthy Communities”^11^ and reducing heart attacks and strokes. However, given the interindividual variability of response to PM_2.5_ and the absence of randomized clinical trials demonstrating the effectiveness of interventions (such as personal protective equipment, in home air purifiers, and behavioral or pharmacological approaches for blocking the adverse cardiovascular events), CDC, *Million Hearts 2027*, and other professional medical organizations do not provide guidance on specific personal interventions that patients may take to reduce PM_2.5_ exposure and cardiovascular disease.

The American Heart Association Scientific Statement “Personal-Level Protective Actions Against Particulate Matter Air Pollutant Exposure”^12^ may aid health professionals in understanding and navigating this conundrum with a conceptual framework to guide personal actions for reduced PM_2.5_ exposure. These include personal exposure mitigation recommendations, such as using personal N95 respirators, high-efficiency home air filtration systems, indoor portable air cleaners, and automobile air filters and air conditioning. Other behavior mitigation recommendations include avoiding areas with high air pollution or staying indoors with closed windows and modifying physical activity when air pollution levels are high to help individuals reduce their exposure to PM_2.5_.^12^ The authors’ proposal takes a more precautionary health protective approach and provides support for specific personal actions to reduce exposure to PM_2.5_ as long as such actions are proportionate to the risk of the patient, relatively inexpensive, accessible, easily implemented, and do not impart collateral risks.^12^ The findings of studies mentioned here and others underscore the importance of increasing awareness in health-care professionals, and patients at higher risk about the possible health effects of PM_2.5_ exposure and speak to the importance of further research including randomized controlled clinical trials of interventions to reduce exposure in higher risk individuals.^13^

The focus of *JACC: Advances* is to examine new and emerging fields of cardiology. Strictly speaking “Environmental Cardiology” is not new, as the name was first used by Dr Aruni Bhatnagar^14^ in 2004 to describe the rapid knowledge expansion related to the association between environmental factors and cardiovascular disease. The field has contributed substantively to the clinical, epidemiological, and toxicological human health data used for regulatory action to improve air quality. However, the translation of the health data into actionable clinical and personal guidance to reduce exposures and associated risks has lagged. Nevertheless, researchers within “Environmental Cardiology” are already tackling the next wave of environmental concerns. They are currently exploring the potential implications of climate change (eg, wildfire smoke, drought, and extreme heat and cold), heavy metals, and countless chemicals of concern (eg, PFAS [Per- and polyfluoroalkyl substances]), as well as their interaction with social determinants of health (eg, environmental justice, disparities in exposure, and risks of health outcomes), and the salutary effects of the characteristics of the built environment on health.

Indeed, the future translation of “Environmental Cardiology” into clinical practice appears bright as the field is poised for greater growth that will lead to the scientific breakthroughs needed to inform policies, identify effective exposure reduction interventions, and ultimately reduce cardiovascular risks and protect public health. Kaufman et al^15^ recently addressed environmental stressors (such as sources of pollutants) that impart a cardiovascular burden and offered mitigating factors (such as green space and parks, increased renewable energy usage, local food production/healthy food consumption, person-centric built environment, and less resource-intensive health care) that could be amenable to policy related actions. It is also important to note that many of the proposed actions suggested by Kaufman et al^15^ are also actions that have historically fallen along racialized boundaries created by historical policies (such as red-lining), which lead to environmental features like increased air pollution exposures amongst minority communities and lowered access to beneficial environmental features, such as green space and healthy food goods.

A recent study by Kumar et al^16^ noted that despite similarities in long-term follow-up and medications, Black patients undergoing percutaneous coronary intervention had worse outcomes than other patients as related to mortality and major adverse clinical events. The potential role of environmental justice,-ie, the inequitable distribution of poor environmental conditions by demographic factors such as race-cannot be overlooked in these disparities and thus the need for clinical, community, and policy interventions to address it cannot be overstated. Access to clinical cohorts and electronic health databases are permitting more informative epidemiological studies assessing the impact of environmental exposures on cardiovascular risk factors, clinical cardiovascular events, and health-care utilization. These important studies achieve the size, clinical phenotyping, and demographic detail necessary to address many open questions in Environmental Cardiology. The consistent observations of these studies-that air pollution is a modifiable risk factor particularly for those with cardiovascular disease-is bolstered by experimental evidence in animal toxicology studies and controlled human exposure studies and underscores the apparent opportunity to improve cardiovascular outcomes through reductions in ambient PM_2.5_ concentrations.

Today, evidence supports environmental quality (both chemical and non-chemical) as an important determinant of cardiovascular health and clinical outcomes. Environmental Cardiology as a field has grown and is now positioned to conduct research and offer specific preventative actions to mitigate the adverse cardiovascular health effects of exposure to air pollutants such as PM_2.5_. Developing a specific framework apart from the regulatory system to address modifiable environmental risks of cardiovascular disease and its sequelae will require the integration, co-operation, and collaboration of multidisciplinary stakeholders. These actions, along with continued work by the state governments to attain the NAAQS for PM have the potential to reduce cardiovascular and stroke morbidity and mortality. Randomized controlled trials of individual-level interventions to reduce PM_2.5_ exposure are essential to guide appropriate recommendations for approaches proven to improve cardiovascular and cerebrovascular outcomes in those at higher risk from PM_2.5_ exposure. The study by Motairek et al^1^ further informs the well-established relationship between PM_2.5_ exposure and adverse cardiovascular outcomes specifically in a population of individuals with elevated susceptibility. The ability to identify such populations and provide them with clear and consistent health information and actions to take, to reduce or mitigate PM_2.5_ exposures will be instrumental to more fully protecting public health from the detrimental impacts of poor air quality.
